# Job vacancies at the European Centre for Disease Prevention and Control (ECDC)

**DOI:** 10.2807/1560-7917.ES.2021.26.37.2109161

**Published:** 2021-09-16

**Authors:** Eurosurveillance editorial team

**Affiliations:** 1 European Centre for Disease Prevention and Control (ECDC), Stockholm, Sweden

The European Centre for Disease Prevention and Control (ECDC) invites applications for the following positions:

 

**Expert Antimicrobial Resistance and Healthcare-Associated Infections**
The application deadline expires on 14 Oct 2021.

 

**Expert Microbial Safety of Substances of Human Origin**
The application deadline expires on 25 Oct 2021.

 

For more information, please visit the ECDC website: 

https://ecdc.europa.eu/en/work-us/vacancies?f%5B0%5D=deadline_date%3A1

